# Baricitinib treatment of severe chronic hand eczema: Two case reports

**DOI:** 10.1111/cod.14039

**Published:** 2022-01-21

**Authors:** Fieke M. Rosenberg, Laura Loman, Marie L.A. Schuttelaar

**Affiliations:** ^1^ Department of Dermatology University Medical Center Groningen, University of Groningen Groningen The Netherlands

**Keywords:** baricitinib, hand dermatitis, hand eczema, hyperkeratotic hand eczema, atopic hand eczema, JAK‐inhibitor, treatment

Janus Kinase (JAK) inhibitors target the JAK and signal transducer and activator of transcription (STAT) signaling pathway that modulates T helper (Th)1, Th2, Th17, and Th22 signaling and is involved in many immune‐mediated diseases. Baricitinib, an oral selective JAK‐1, and ‐2 inhibitor, has recently been approved for adults with moderate‐to‐severe atopic dermatitis (AD).^1^ The effect of baricitinib on chronic hand eczema (CHE) has not yet been described. Here we present two cases of CHE successfully treated with baricitinib.

## CASE REPORTS

### Case 1

A 52‐year‐old man, who works as a builder, was diagnosed with severe hyperkeratotic CHE for 6 years. He has a history of asthma in childhood (not classified), allergic rhino‐conjunctivitis, and no history of AD. Routine patch testing was performed and the results were negative. His CHE did not improve, despite avoidance of work‐related irritants. Treatment history included ultra‐potent topical corticosteroids, alitretinoin, acitretin, cyclosporine, apremilast (all discontinued due to inefficacy), and methotrexate (discontinued due to side effects).

### Case 2

A 55‐year‐old woman, who works as an administrator, had severe AD with concomitant severe atopic CHE for 5 years. She has a history of allergic asthma, allergic rhino‐conjunctivitis, food allergies, and kerato‐conjunctivitis. She had no relevant exposure to irritants, and there were no contact allergies with clinical relevance to the hands. Treatment history included ultra‐potent topical corticosteroids, topical calcineurin inhibitors, prednisolone, methotrexate, cyclosporine, omalizumab (all discontinued due to inefficacy), and dupilumab (discontinued due to side effects).

Both patients were treated with baricitinib (4 mg tablet once per day), because Case 1 had severe refractory CHE and Case 2 had AD with concomitant severe CHE, that had great impact on their quality of life.

**FIGURE 1:**
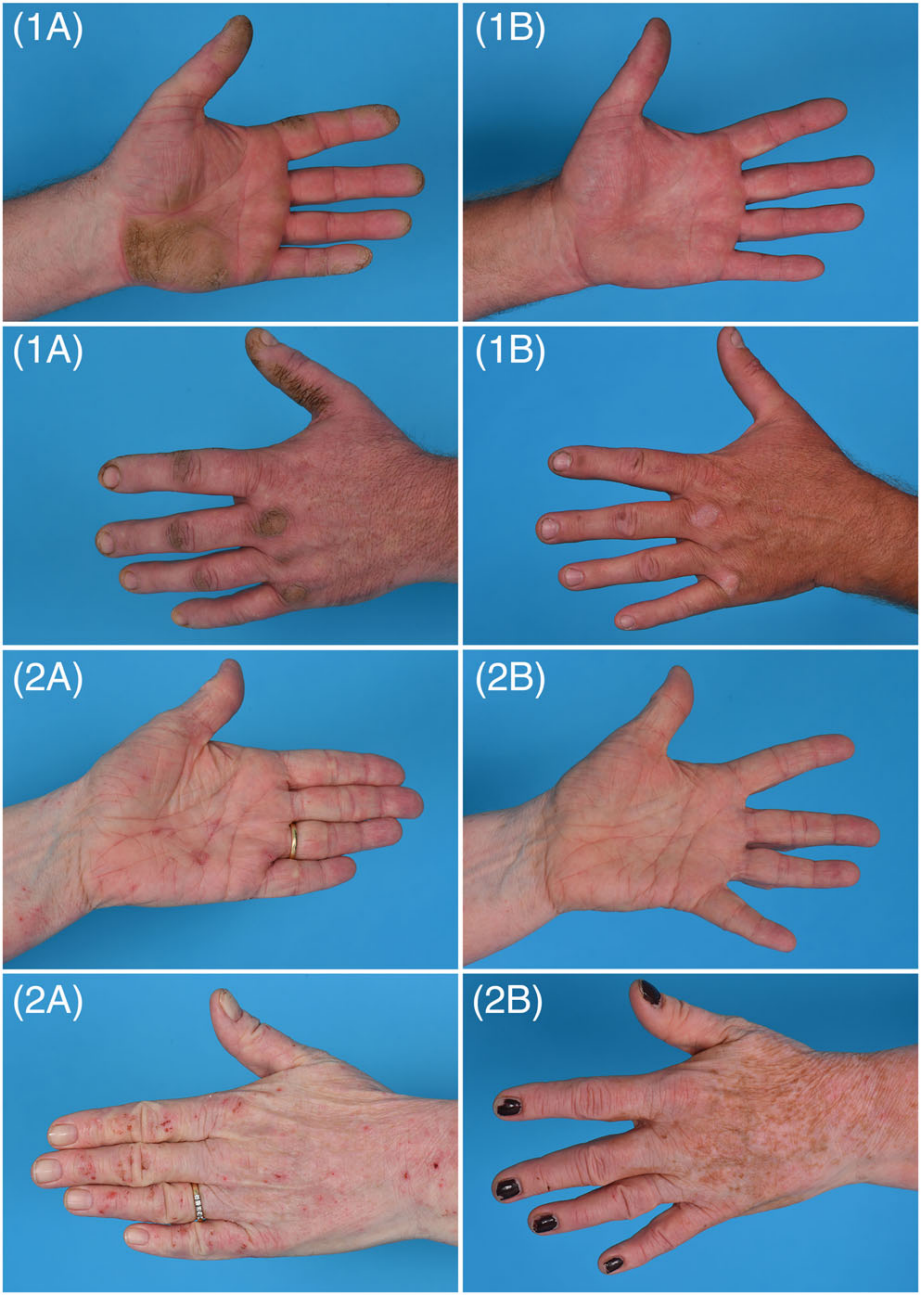
Clinical improvement of two cases of severe chronic hand eczema after 16 weeks of baricitinib treatment. Case 1: severe hyperkeratotic chronic hand eczema; improvement from 1(**A**) baseline to 1(**B**) week 16. Case 2: Severe atopic chronic hand eczema, improvement from 2(**A**) baseline to 2(**B**) week 16

Case 1 presented at baseline with ‘severe’ CHE according to the photographic guide^2^ and a hand eczema severity index (HECSI)^3^ score of 55 (severe^4^). After 16 weeks of treatment, the CHE was improved to “almost clear” and the HECSI score to 4 (Figure 1). The patient’s quality of life improved from “strongly impaired” to “not at all impaired” based on the Quality of Life in Hand Eczema Questionnaire (QOLHEQ).^5^ Emollients were continued during bariticinib treatment. Baricitinib was well‐tolerated as no side effects occurred.

Case 2 presented at baseline with “severe” CHE and a HECSI score of 47 (severe^4^). Baricitinib dosage was tapered to 2 mg per day at 12 weeks on request of the patient due to the good effect. After 16 weeks of treatment, the CHE was improved to “almost clear” and the HECSI score to 8 (see Figure 1). Her quality of life improved from “moderately impaired” to “not at all impaired” based on the QOLHEQ. Her AD was improved from “severe” to ‘moderate’ at 16 weeks of treatment based on a 5‐point Investigator Global Assessment scale for AD.^6^ Emollients were continued, and a potent topical corticosteroid was used to a limited extent (<10 g per week for AD and CHE) during baricitinib treatment.

However, she discontinued barcitinib because of a bacterial corneal ulcer at 16 weeks. The culture of the corneal ulcer was positive for *Staphylococcus aureus*, while the culture of her scleral lens was negative. (See Supplement 1, Table S1 for all patient characteristics and outcome measures.)

## DISCUSSION

Two patients with CHE were successfully treated with baricitinib as demonstrated by a decrease in clinical symptoms and improvement in quality of life. The pathogenesis of CHE is not fully elucidated, although Th1, Th2, Th17, and Th22 signaling seem to play a role. The JAK–STAT signaling pathway is involved in the signaling of several cytokines that have been shown to play a role in the pathophysiology of atopic CHE, such as interleukin (IL) 4, IL‐13, IL‐22 and IL‐31,^7^, ^8^ and hyperkeratotic CHE, such as IL‐1, IL‐17, and interferon‐gamma.^8^ Considering that JAK‐inhibitors target several cytokine pathways instead of one single pathway, we hypothesized that this could be an effective therapy for the majority of CHE patients.^1^, ^7^

Our hypothesis was strengthened by previous literature on three different JAK‐inhibitors that showed promising efficacy on CHE. Recently, two phase 3 trials with upadacitinib, an oral JAK‐1 inhibitor, presented significant improvements in mild‐to‐severe CHE in patients with moderate‐to‐severe AD compared to placebo at 16 weeks.^9^ A phase 2b trial with gusacitinib, an oral dual pan‐JAK/spleen tyrosine kinase (SYK)‐inhibitor, reported dose‐dependent improvements of moderate‐to‐severe CHE up to 16 weeks compared to placebo.^10^ Further, a phase 2a trial with delgocitinib ointment 30 mg/g, a topical pan‐JAK inhibitor, showed significant improvements in mild‐to‐severe CHE compared to placebo at 8 weeks.^11^ The subsequent phase 2b trial with delgocitinib cream presented a significant dose–response relationship in efficacy compared to placebo in mild‐to‐severe CHE at 16 weeks. The ongoing phase 3 trial investigates the efficacy and safety of delgocitinib cream (20 mg/g) up to 16 weeks.^12^

In the near future, patients with moderate‐to‐severe refractory CHE, regardless of subtype, may benefit from treatment with JAK‐inhibitors. Further research is necessary to investigate the efficacy and safety profile on a larger scale.

## CONFLICT OF INTEREST

M.L.A. Schuttelaar received consultancy fees from Sanofi‐Genzyme and Regeneron Pharmaceuticals; and is an advisory board member for Sanofi‐Genzyme, Regeneron Pharmaceuticals, Pfizer, LEO Pharma, Lilly. The other authors have no other conflicts to declare.

## AUTHOR CONTRIBUTIONS

**Marie L.A. Schuttelaar:** Conceptualization (lead); investigation (supporting); methodology (equal); supervision (lead); writing – review and editing (equal). **Fieke M. Rosenberg:** Conceptualization (equal); data curation (lead); formal analysis (lead); investigation (lead); methodology (equal); project administration (lead); visualization (lead); writing original draft (lead); writing ‐ review and editing (supporting). **Laura Loman:** Conceptualization, (equal); data curation (supporting); methodology (equal); supervision (supporting); writing – review and editing (equal). All authors read and approved the final manuscript.

## Supporting information


**Table S1** Patient characteristics and outcome measures.
