# Supplementary material for: Baricitinib treatment of severe chronic hand eczema: Two case reports
Source: Contact Dermatitis. 2022 Jan 21;86(5):419–21. doi: 10.1111/cod.14039 (PMC9306776; doi:10.1111/cod.14039)
Supplement: Supplementary file 1 — Table S1 Patient characteristics and outcome measures. [file COD-86-419-s001.docx]

**Online supplemental material**

**Baricitinib treatment of severe chronic hand eczema: two case reports**

Fieke M. Rosenberg, Laura Loman, Marie L.A. Schuttelaar

**Table S1. Patient characteristics and outcome measures**

|  | Case 1 | Case 2 |
| --- | --- | --- |
| Age^†^ | 51 | 55 |
| Sex | Male | Female |
| Disease duration in years | 6 | 5 |
| Occupation | Builder | Administrator |
| Smoking status^‡^ | 0 | 0 |
| Atopic comorbidities:  Atopic dermatitis   Asthma  Allergic rhinitis | - **+** + | +  +  + |
| Total IgE^§^ | - | + |
| Specific IgE inhalant allergens^¶^ | + | + |
| Relevant positive patch test results | - | - |
| Irritant contact dermatitis (wet work criteria)^1^ | + | - |
| Treatment history:   - Topical ultra-potent corticosteroid - Topical calcineurin inhibitors - Alitretinoin - Acitretin - Cyclosporine - Methotrexate - Prednisolone - Dupilumab - Apremilast - Omalizumab | +  -  + + + + - - + - | +  +  -  -  +  +  +  +  -  + |
| Photographic guide^2^  Baseline  Week 4  Week 16 | Severe  Severe  Almost clear | Severe  Moderate  Almost clear |
| HECSI^3^  Baseline  Week 4  Week 16 | 55  22  4 | 47  11  8 |
| QOLHEQ^4^  Baseline  Week 4  Week 16 | 67  29  2 | 61  19  8 |

- = no, + = yes, †= years, ‡ = in pack-years; defined as twenty cigarettes smoked per day for one year, § = above 115 kU/L, ¶= above 0.34 kU/L.

Abbrevations: IgE = Immunoglobulin E, HECSI = Hand Eczema Severity Index^3^, QOLHEQ^4^ = Quality of Life in Hand Eczema Questionnaire, kU/L = kilo units per liter.

1. Oosterhaven JAF, Flach PA, Bültmann U, Schuttelaar MLA. Presenteeism in a Dutch hand eczema population—a cross-sectional survey. *Contact Dermatitis*. 2018;79(1):10-19. doi:10.1111/cod.12993

2. Coenraads PJ, Van Der Walle H, Thestrup-Pedersen K, et al. Construction and validation of a photographic guide for assessing severity of chronic hand dermatitis. *Br J Dermatol*. 2005;152(2):296-301. doi:10.1111/j.1365-2133.2004.06270.x

3. Held E, Skoet R, Johansen JD, Agner T. The hand eczema severity index (HECSI): a scoring system for clinical assessment of hand eczema. A study of inter- and intraobserver reliability. *Br J Dermatol*. 2005;152(2):302-307. doi:10.1111/j.1365-2133.2004.06305.x

4. Oosterhaven JAF, Ofenloch RF, Schuttelaar MLA. Validation of the Dutch Quality of Life in Hand Eczema Questionnaire (QOLHEQ). *Br J Dermatol*. 2020;183(1):86-95. doi:10.1111/bjd.18558
